# Genito-Urinary Surgery

**Published:** 1905-08-12

**Authors:** 


### Progress in Medicine and Surgery.

### GENITO-URINARY SURGERY.

(*Continued from page* 328.)

**Pyelitis.**—Howard A. Kelly (Baltimore)[3] has written on the treatment of pyelitis. The condition in its earlier stages is still rarely recognised or regarded seriously enough. It may get well spontaneously, or remain stationary for long periods, or grow worse and worse. Treatment must be based upon the cause in each case. In one case the bacillus typhosus was found, though it was thirty years previously that the patient had had typhoid. Some cases depend upon gastro-intestinal disturbance—such as obstipation—and the relief of this will do much towards the relief of the pyelitis. Even sucklings sometimes have pyelitis. The unsuspected presence of a hydro-nephrosis of low grade is not infrequently the cause of pyelitis. In such cases the vermiform appendix, or the right Fallopian tube and ovary, have been removed without benefit. The pain may be traced to the kidney by introducing a renal catheter to the renal pelvis and injecting fluid when an attack of pain is brought on of the same character as that about which the patient has complained. A dilated renal pelvis is prone to catch a low-grade inflammation if the general health becomes depressed, or if there is a focus of suppuration elsewhere. In these cases an important sign of the pyelitis is the escape of a thick sediment from the pelvis of the kidney at the end of catheterisation of the ureter. Débris of this kind left in the renal pelvis perpetuate infection indefinitely. Sometimes a single catheterisation will lead to permanent relief. If a mobile kidney is pyelitic, it is advisable to relieve the pyelitis before fixing the kidney. In another class of cases of pyelitis the cause is cystitis, often of mild degree, affecting the base of the bladder and causing stricture of the ureteral orifice, with more or less stasis of urine above the stricture. Sometimes a pyelitis follows the removal of an ovarian or fibroid tumour, which has been pressing on the ureter. The crippled kidney catches infection after the operation. Extensive dense strictures of the lower end of the ureter may be associated with tuberculosis of the kidney, and the condition is often combined with secondary pyogenic infection. The vermiform appendix may become adherent to, and compress the ureter and lead to pyelitis. In the treatment of pyelitis the essential thing is to remove the cause. The following methods of treatment are available:—rest with the administration of diluents and drugs such as urotropin and salol; the local treatment of cystitis; incision or dilatation of a stricture of a ureteral orifice; catheterisation of the ureter and renal pelvis or irrigation of the same; instillations into the renal pelvis; and nephrotomy, nephrostomy, or nephrectomy. The author cites cases in which some of the more aggressive methods of treatment mentioned above were successfully employed. He regards mild pyelitis as a most important, common, and yet often unrecognised affection. A mild pyelitis may end in gross destructive renal disease. L. W. Allen[5] records a case of chronic pyelitis due to infection with the bacillus coli communis, which was cured by a nephrotomy followed by irrigations through the kidney into the bladder. The symptoms closely resembled those of renal tuberculosis, for which in fact the case was mistaken. The patient was a woman aged 40 who had been ill for two years. The stomach was disordered, vomiting being frequent, and micturition was painful and difficult. The right kidney was large and tender and pus was obtained from its pelvis with the ureteral catheter, and the patient was much emaciated. After the nephrotomy and washing out through a drainage tube which passed into the renal pelvis, through the cortex, a rapid recovery ensued.

[3] Birm. Med. Rev., March 1905, p. 127. [4] Med. Rec., April 8, 1905, p. 521. [5] Amer. Jour. Med. Sci., March 1905, p. 447.

(*To be continued.*)

